# Correction: Yu et al. Antimicrobial Resistance of *Escherichia coli* for Uncomplicated Cystitis: Korean Antimicrobial Resistance Monitoring System. *Antibiotics* 2024, *13*, 1075

**DOI:** 10.3390/antibiotics14030234

**Published:** 2025-02-25

**Authors:** Seong Hyeon Yu, Seung Il Jung, Seung-Ju Lee, Mi-Mi Oh, Jin Bong Choi, Chang Il Choi, Yeon Joo Kim, Dong Jin Park, Sangrak Bae, Seung Ki Min, KAUTII Investigators

**Affiliations:** 1 Department of Urology, Chonnam National University Hospital, Chonnam National University Medical School, Gwangju 61469, Republic of Korea; domer12@hanmail.net; 2 Department of Urology, The Catholic University of Korea, St. Vincent’s Hospital, Suwon 16247, Republic of Korea; lee.seungju@gmail.com; 3 Department of Urology, Korea University Guro Hospital, Seoul 08308, Republic of Korea; mamah77@paran.com; 4 Department of Urology, Bucheon St. Mary’s Hospital, College of Medicine, The Catholic University of Korea, Seoul 14647, Republic of Korea; c-sparrow@hanmail.net; 5 Department of Urology, Hallym University Dongtan Sacred Heart Hospital, College of Medicine, Hallym University, Hwaseong 18450, Republic of Korea; choicog@gmail.com; 6 Department of Urology, Daegu Fatima Hospital, Daegu 41199, Republic of Korea; winewiner85@gmail.com; 7 Department of Urology, Dongguk University College of Medicine, Gyeongju 38066, Republic of Korea; parkdj0510@gmail.com; 8 Department of Urology, Uijeongbu St. Mary’s Hospital, College of Medicine, The Catholic University of Korea, Seoul 11765, Republic of Korea; robinbae97@catholic.ac.kr; 9 Department of Urology, Goldman Urologic Clinic, Seoul 05510, Republic of Korea; msk0701@hanmail.net

## Error in Figure

In the original publication [1], there was a mistake in Figure 1c as published. In Figure 1c, recurrence and non-recurrence were swapped in the legend. The corrected Figure 1 appears below. The authors state that the scientific conclusions are unaffected. This correction was approved by the Academic Editor. The original publication has also been updated.

## Figures and Tables

**Figure 1:**
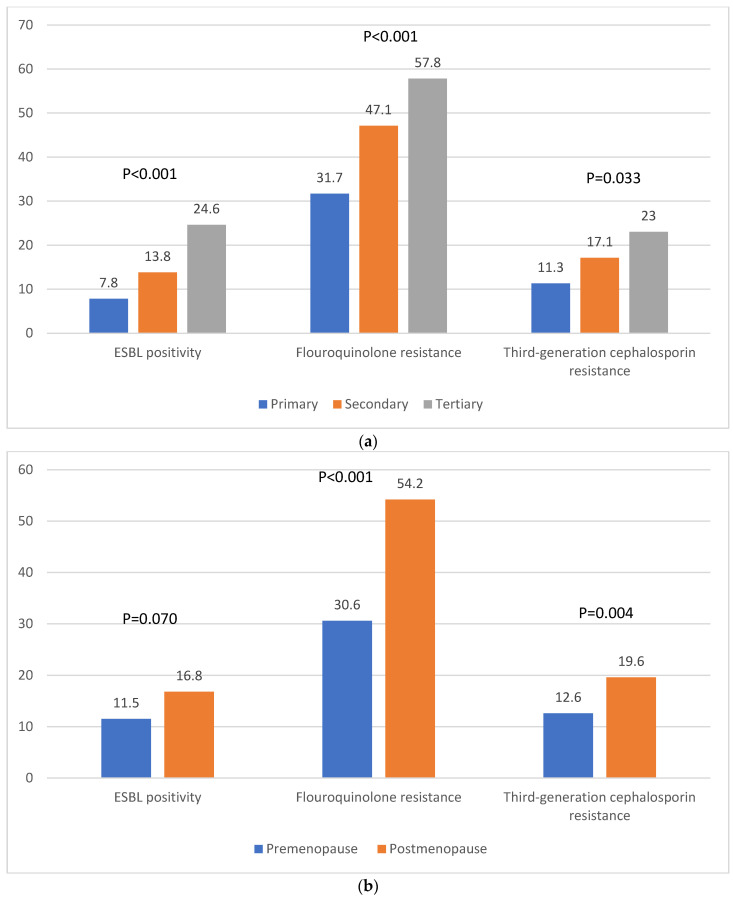

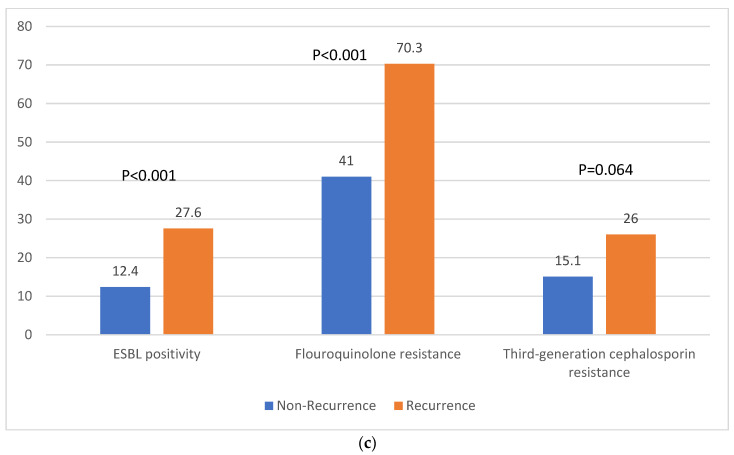
ESBL positivity, fluoroquinolone resistance, and third-generation cephalosporin resistance of *E. coli* according to clinical factors. (**a**) Hospital type, (**b**) menopausal status, and (**c**) recurrence of infection.

## References

[1] Yu S.H., Jung S.I., Lee S.-J., Oh M.-M., Choi J.B., Choi C.I., Kim Y.J., Park D.J., Bae S., Min S.K. (2024). Antimicrobial Resistance of *Escherichia coli* for Uncomplicated Cystitis: Korean Antimicrobial Resistance Monitoring System. Antibiotics.

